# Directional Reflective Surface Formed via Gradient-Impeding Acoustic Meta-Surfaces

**DOI:** 10.1038/srep32300

**Published:** 2016-08-26

**Authors:** Kyungjun Song, Jedo Kim, Shin Hur, Jun-Hyuk Kwak, Seong-Hyun Lee, Taesung Kim

**Affiliations:** 1 Department of Nanoconvergence Systems, Korea Institute of Machinery and Materials, 156 Gajeongbuk-Ro, Daejeon, 305-343, Republic of Korea; 2 Department of Mechanical System Design Engineering, Hongik Univ. 94 Wausan-ro, Mapo-gu, Seoul 121-791, Seoul, Republic of Korea; 3 Department of System Dynamics, Korea Institute of Machinery and Materials, 156 Gajeongbuk-Ro, Daejoen, 305-343, Republic of Korea; 4 Department of Mechanical Engineering, Ulsan National Institute of Science and Technology (UNIST), 50 UNIST-gil, Ulsan 44919,, Republic of Korea

## Abstract

Artificially designed acoustic meta-surfaces have the ability to manipulate sound energy to an extraordinary extent. Here, we report on a new type of directional reflective surface consisting of an array of sub-wavelength Helmholtz resonators with varying internal coiled path lengths, which induce a reflection phase gradient along a planar acoustic meta-surface. The acoustically reshaped reflective surface created by the gradient-impeding meta-surface yields a distinct focal line similar to a parabolic cylinder antenna, and is used for directive sound beamforming. Focused beam steering can be also obtained by repositioning the source (or receiver) off axis, i.e., displaced from the focal line. Besides flat reflective surfaces, complex surfaces such as convex or conformal shapes may be used for sound beamforming, thus facilitating easy application in sound reinforcement systems. Therefore, directional reflective surfaces have promising applications in fields such as acoustic imaging, sonic weaponry, and underwater communication.

Excluding digital signal processing technology such as phase array systems^1^,^2^ or modulated ultrasound speakers^3^,^4^, several different methods exist for achieving directive sound beamforming, e.g., those using acoustic horns^5^, phononic crystals (PCs)^6^,^7^,^8^,^9^,^10^,^11^,^12^,^13^,^14^, and acoustic metamaterials^15^. For example, the most well-known type of acoustic horn is a megaphone or bullhorn, which is a conical device used to amplify and direct sound in a particular area by restricting the sound wave dispersal^5^. However, to achieve any degree of directivity, the dimensions of such devices must be several times larger than the sound wavelength, *λ*, which renders them quite large and bulky. PCs can also be used to achieve high directivity through the manipulation of acoustic surface waves^6^,^7^,^8^, resonant cavity states^9^,^10^, or band-edge states^11^,^12^,^13^,^14^. However, PC-based methods typically result in narrow bandwidths and require device scales larger than the *λ* of interest. As an alternative, acoustic metamaterials provide the means to manipulate sound waves to an extraordinary extent, while having the capacity for miniaturization^16^,^17^,^18^,^19^,^20^,^21^,^22^,^23^,^24^. One important application of acoustic metamaterials is the directive sound antenna. A recent attempt at constructing such a device, which involved an artificially textured impedance surface with a pressure release boundary, utilized a periodic array of identical Helmholtz resonators^15^. Although subwavelength in scale, the periodic element array achieved relatively low directivity, because of the nature of the dipole radiation.

In the field of electromagnetics, the parabolic antenna yields the highest gains, *G*, thus providing the narrowest beamforming of any antenna type^25^. Such high gain (*G*) originates from the feed antenna located at the focal point of the parabolic reflection dish, which creates a geometrically induced reflection phase gradient. Thus, a parabolic antenna transmits signals over greater distances than conventional methods such as horns. Although this concept can be easily applied to acoustics, a curved reflector dish that is required to be quite bulky must be incorporated in order to induce reflection phase differences. Nevertheless, the development of acoustic metamaterials has alleviated many spatial restrictions. These metamaterials employ subwavelength structures, and it is now possible to design flat meta-surfaces comprised of individual components that induce progressive changes in the reflection phase of an acoustic signal using subwavelength resonators^16^,^26^. These flat meta-surfaces, if designed carefully, can then induce parabolic or hyperboloidal phase gradients (similar to their electromagnetic counterparts), which geometrically induce the same gradient. In addition, it should be possible to employ flexible or conformal meta-surfaces (if realized) in directive sound beamforming, because of the resultant generation of acoustically reshaped phase reflection along these complex surfaces.

Here, we introduce a new type of directional sound antenna that utilizes an acoustic metamaterial surface composed of progressively varying effective impedance structures. These structures cause the flat surface to behave as a parabolic cylindrical antenna. We begin by describing the basic principles behind the achievement of directive beamforming using the focal line of a planar meta-surface. Then, we present theoretical and numerical results predicting the behaviour of the gradient-impeding meta-surface, which consists of a periodic array of Helmholtz resonators that function as both a reflector and a phase shifter. We also present experimental results showing the highly directive sound pressure level (SPL) obtained for sound waves originating from a faraway source (plane wave source) and reflected from the meta-surface. Next, we demonstrate that the acoustic meta-surface proposed in the study can be used for a system that focuses sound at an oblique angle if the microphone is positioned off axis. Besides the directional sound receiver, not only a flat meta-surface, but also a convex meta-surface combined with omnidirectional sound sources can act as a directional sound source.

## Results

### Directive Sound Antenna Composed of Artificially Designed Acoustic Meta-surfaces

**Figure 1:**
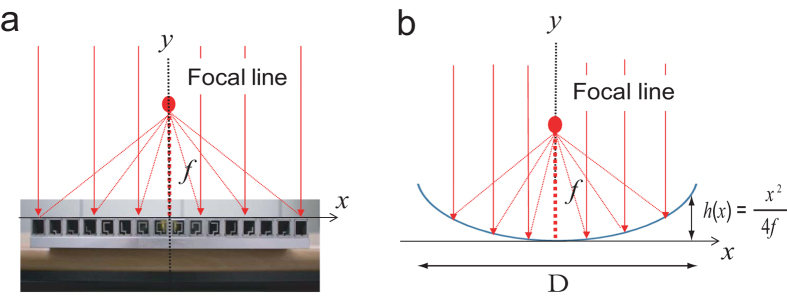
Basic design concept for planar-type sound antenna with curved reflection phase *Φ*(*x*). (**a**) Conceptual figures of planar directional surface with symmetric phase gradient d*Φ*(*x*)/d*x* with respect to the origin. Such surfaces can be obtained using space elements with varying degrees of coiling positioned along the planar surface. The surface can then be used to form a distinct focal line located directly above and at a distance *f* from the origin, as shown in the figure. The structure is invariant in the z direction. (**b**) Conceptual schematic of parabolic antenna, having an identical focal line to the planar meta-surface.

Figure 1 illustrates the basic design concept of a highly directive sound antenna, for which the reflection phase, *Φ*(*x*), is manipulated along an artificially textured surface. This approach is analogous to the design concept of the antenna’s electromagnetic counterpart, which exploits geometrically induced phase differences induced using parabolic reflectors. We consider a perfectly reflecting planar surface that provides gradual phase variation of the reflecting sound waves along the surface. If this surface is designed such that a positive (d*Φ*(*x*)/d*x* > 0) and negative (d*Φ*(*x*)/d*x* < 0) reflection phase gradient are simultaneously achieved in the positive and negative *x* directions, respectively, the focal line of a normally incident beam will then be located at a distance *f* directly above the line of origin, as shown in Fig. 1(a) (*f* is the focal length).

At the focal line, the sound beam is expected to be highly directional; therefore, a highly directive detector using a relatively small omnidirectional receiver can be realized. Although several methods of tailoring the d*Φ*(*x*)/d*x* of a planar surface exist (e.g., linear, conical, or exponential), a design that generates a hyperboloidal *Φ*(*x*) profile from the planar surface^26^ is essential; i.e., we require *Φ* 
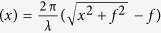
. Such a profile allows us to achieve a high gain (*G*) and high directivity, similar to those obtained for a parabolic reflector with a geometrical curved surface *h*(*x*) = *x*^2^/4*f*, as illustrated in Fig. 1(b). On the other hand, if an obliquely incident sound wave is projected onto the meta-surface, the defocused line is expected to be located some distance away from the axis of origin. Such characteristics enable an omnidirectional receiver located above the axis of origin to yield high directivity for a faraway sound source.

### Reflection Phase Obtained for Acoustic Meta-surface with Gradient Impedance

Recently, acoustic wave-front engineering has been achieved utilizing a high refractive index, which can be tailored through the use of labyrinthine units with subwavelength thickness^16^,^26^,^27^,^28^,^29^,^30^,^31^. These labyrinthine-type structures are described as “high refractive media” for waves propagating parallel to the path direction, and “hard walls” for waves propagating perpendicular to the path direction, thus yielding the anisotropic density tensor. Here, we tune the acoustic impedance by artificially texturing the acoustic surface in order to manipulate the acoustic *Φ*(*x*)^15^,^32^, similar to the case of a high-impedance electromagnetic surface^33^,^34^. These textured surfaces with locally resonant subwavelength elements have an important distinction from the labyrinthine-type surfaces in that they create an effectively homogeneous medium^35^,^36^. Thus, these textured surfaces provide an efficient means of controlling acoustic scattering on complex surfaces.

**Figure 2:**
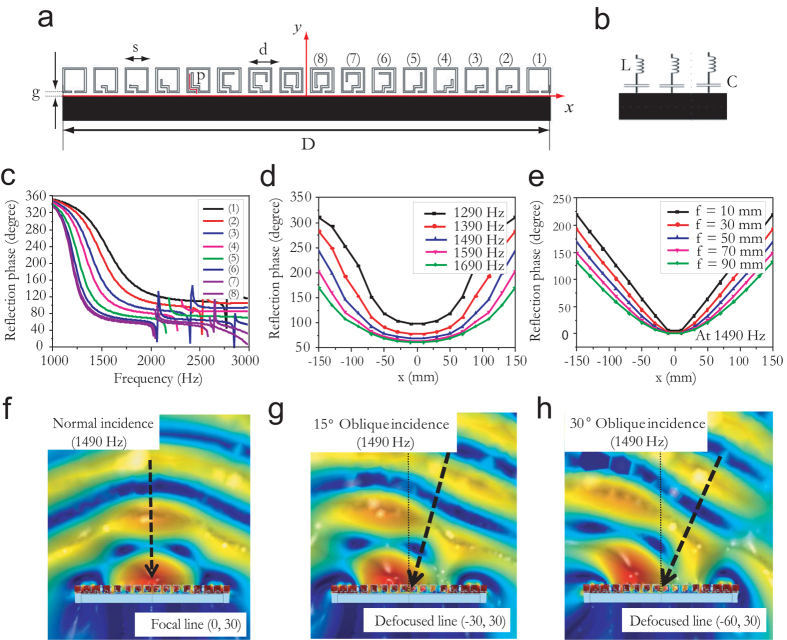
Directive sound antenna design based on acoustic metamaterials mimicking parabolic cylindrical antenna. (**a**) Schematic of acoustic meta-surface consisting of array of Helmholtz resonators with varying coiled path lengths, *p* (Sample 1, number of resonators on each side, *N* = 8). The subwavelength width, *s*, subwavelength separation, *d*, gap, *g*, and total length, *D*, are fixed to 15, 20, 2 and 317.5 mm, respectively, while *p* is adjusted to 1, 5.5, 9, 12.5, 16.5, 24, 31, and 36 mm. The structure is invariant in the z direction. (**b**) Series LC circuit model for resonators comprising the meta-surface, which have minimum impedance for the reflection phase, *Φ*(*x*) = *π*, at the resonance frequency. (**c**) Calculated *Φ*(*x*) for resonators having varying *p*, which comprise the meta-surface. (**d**) Calculated *Φ*(*x*) profile for meta-surface at various incident sound frequencies. (**e**) Calculated parabolic profile of *Φ*(*x*) at 1490 Hz. The profile follows the relationship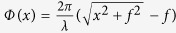, where *λ* is the wavelength and *f* is the focal length. (**f**–**h**) Sound pressure level profiles for normally, 15° obliquely, and 30° obliquely incident plane waves at 1490 Hz resulting in focal line at approximately (0, 30) and defocused lines at approximately (−30, 30) and (−60, 30), respectively. (All positions are in millimetres).

In order to establish a parabolic d*Φ*(*x*)/d*x* along the meta-surface (as shown in Fig. 1), we utilize a periodic array of Helmholtz resonators with subwavelength width, *s*, subwavelength separation, *d*, and various values for the coiled path length, *p*, as depicted in Fig. 2. Figure 2(a) is a schematic of one sample (Sample 1), where the total length, *D*, is 317.5 mm and the number of resonators symmetrically located on either side of the origin, *N*, is 8. Schematics of Sample 2 (*N* = 6; *D* = 237.5 mm) and Sample 3 (*N* = 4; *D* = 157.5 mm) are shown in Supplementary Fig. S1.

The array comprised of these resonators can be considered as an effective medium, as the value of *d* (between the resonators) is significantly smaller than the *λ* of interest. Each Helmholtz resonator has a unique *p*; this individuality results in a distinctive impedance and, in turn, *Φ*(*x*). We apply a symmetric impedance variation with respect to the axis of origin, so that the focal line is in line with the origin for normally incident plane wave radiation. Then, the phase of the reflected wave propagating through a medium having the characteristic impedance of air, *η*, and reflecting from a textured meta-surface with surface impedance *Z*_m_ is expressed as


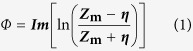


where *η* = *ρc* is the impedance of air (*ρ* ~ 1.3 kg/m^3^ is the density of air) and ln is the natural logarithm.

Under the effective medium theory, the resonators can be considered as an array of effective media, for which the impedances can be modelled as inductor-capacitor (LC; *L* and *C* represent the inductance and capacitance hereafter) resonant circuits in series with lumped impedance, *Z*_*m*_ = *jωL* + *1/jωC*, where *ω* is the frequency and *j* is the imaginary unit. *Z*_*m*_is determined by the properties of the individual resonators, as shown in Fig. 2(b). Note that we ignore the absorption due to the viscosity of air, because of the negligible viscous boundary layer existing at the air-hard wall interface, *l*_vis_ = 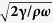, where *γ* ≈ 1.7 × 10^−5^ Pa·s denotes the air viscosity^5^. At *ω* = 1490 Hz, *l*_vis_ ≈ 0.05 mm.

From this model, the impedances of the individual resonators can be tuned to create the desired d*Φ*(*x*)/d*x* through geometric changes to the resonators. The *C* (i.e., acoustic compliance) values can be altered by changing the internal volumes of the resonators, while the *L* (i.e., acoustic mass) values can be tuned by changing the *d*, *g*, *s*, and *p* of the resonators. For example, resonator 1 in Sample 1 (Fig. 2(a)) yields large *C* due to the large internal volume of the cavity, but small *L* due to small *p*, whereas for resonator 8 in Sample 1 (Fig. 2(a)), the opposite is true. Thus, *C* and *L* can be gradually varied through careful design of the cavity inner structure, as shown in Fig. 1(a). Based on the *LC* resonator circuit theory, it can be expected that *Φ*(*x*) varies from 2*π* to *π* from low *ω* to the resonance frequency, *ω*_o_ ≈ 1/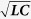, because of the dominance of *C*. In contrast, from *ω*_o_ to high *ω*, *Φ*(*x*) varies from *π* to 0 because of the *L* dominance. Then, for the designed meta-surface shown here, it is possible to realize a full *Φ*(*x*) variation form 0 to 2*π* through modulation of the coiled spaces within the resonators. Figure 2(c) shows the calculated *Φ*(*x*) for the eight different Helmholtz resonators in Sample 1, having varying degrees of *p*. At its *ω*_o_, each Helmholtz resonator satisfies the pressure release boundary condition, because *Φ*(*x*) = π corresponds to the lowest impedance. The radiation bandwidth (BW) of each resonator covering the range of *Φ* = π/2 (90°) − 3π/2 (270°) can be approximately expressed as (see Supplementary Note)


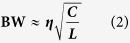


Equation (2) shows the basic design rule used to control the bandwidths of artificial impedance surfaces for an acoustic antenna. For structures with small mass and a large internal cavity volume, the radiation bandwidth is broader, whereas for structures with large mass and a small internal cavity volume, the radiation bandwidth is narrower. Thus, resonator 8 in sample 1, which has high L and small C, provides a narrow usable bandwidth, whereas resonator 1 in the same Sample, having low L and high C, yields a broad usable bandwidth, as confirmed by Fig. 2(c). That figure also shows that almost the full range of phase control is possible with an ultrathin acoustic meta-surface with s + g ≈ *λ*/13 and d ≈ *λ*/11 (at 1490 Hz). Figure 2(d) shows the calculated *Φ*(*x*) along the acoustic meta-surface for various *ω*, and Fig. 2(e) illustrates the reflection surface with the same *D* and a hyperboloidal profile at 1490 Hz, which corresponds to a different *f*. As illustrated, the d*Φ*(*x*)/d*x* of the textured surface at *ω* = 1490 Hz resembles the hyperboloidal profile at *f* = 30 mm. In addition, for p ranging from 1 mm to 36 mm, we conduct the polynomial interpolation to predict the reflection phase *Φ* at 1490 Hz as a function of the path *p* as illustrated in Fig. S3.


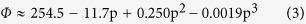


Here the reflection phase *Φ* is in degree and *p* is in mm. This mathematical function leads to reflection phase as a function of desired coiling path *p*, thus providing the design flexibility of acoustic meta-surface.

Figure 2(f–h) illustrate the numerically modelled sound pressure level (SPL) reflected from the acoustic meta-surface for normally, 15° obliquely (at the normal surface), and 30° obliquely incident waves (at the normal surface) for *ω* = 1490 Hz, respectively. When the incident wave impinges in the normal direction, the focal line is located at *f* ≈ (0 mm, 30 mm) from the origin. (Hereafter, all positions are given in millimetres.) For waves impinging at an oblique angle, the defocused line is located at *f* ≈ (−30, 30) and ≈ (−60, 30) for 15° and 30° incidence, respectively. Beside induced phase modulation, beam focusing very close to the meta-surface is affected by near field intensity and diffraction around sharp corners (or edges) of resonators. Nonetheless, the existence of a focal line results in enhanced SPL at a fixed line in space, yielding an interaural level difference (ILD) depending on the angle of incidence, *θ*. This fact allows for convenient identification of the location of a sound source using the polar sound intensity variation information, even when a single omnidirectional microphone is used.

### Directive Sound Receiver based on Acoustic Meta-surface

**Figure 3:**
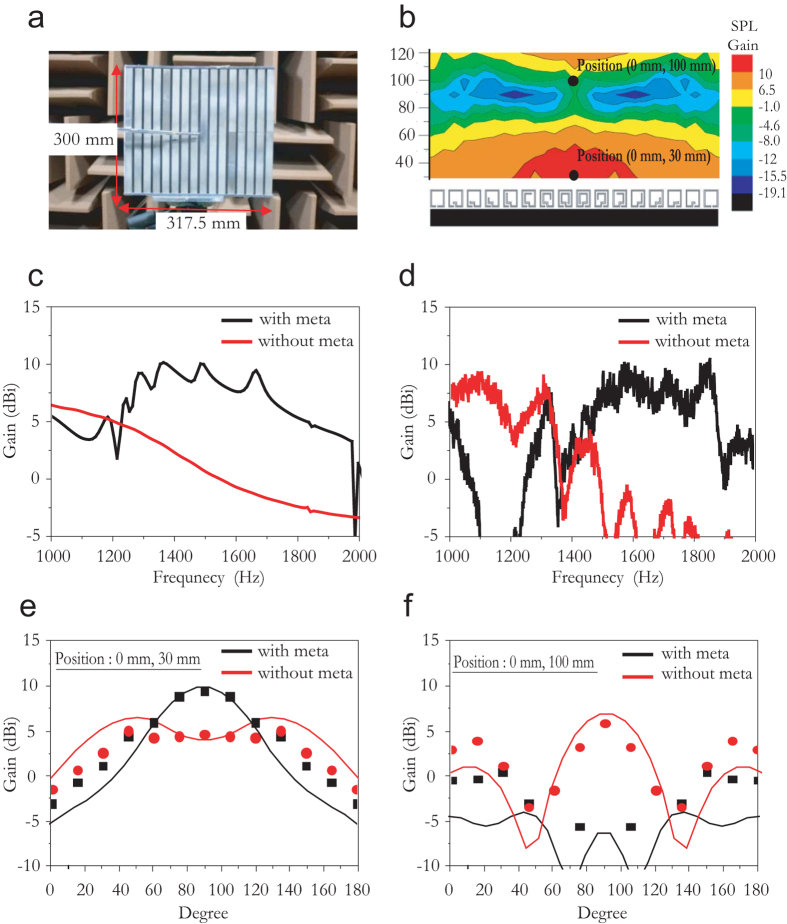
Acoustic antenna in receiving mode based on artificial textured meta-surface mimicking parabolic-cylinder antenna. (**a**) Photograph of prototype meta-surface Sample 1 with subwavelength separation *d* = 20 mm. (**b**) Measured SPL gain contour near acoustic meta-surface located 1.5 m from source speaker with 1490 Hz pure tone. (**c**) Calculated and (**d**) measured sound pressure level (SPL) gain at (0, 30) from 1000 to 2000 Hz for normally incident sound wave. (**e,f**) Calculated (line) and measured (dot) polar SPL gain at (0, 30) and (0, 100) for 1490 Hz incident frequency, respectively. (All positions are in millimetres).

As shown in Fig. 3(a), we experimentally demonstrate a directive acoustic receiver by constructing an acoustic metamaterial with Sample 1 characteristics, having *d* = 20 mm and mimicking a parabolic-cylinder antenna. Figure 3(b) shows the measured acoustic field distribution for a normally incident sound source with *ω* = 1490 Hz. The experimental result is in close agreement with the numerical prediction (Fig. 2f). Here, the SPL of the microphone near the rigid planar structure with (*P*_m_) and without (*P*_r_) the metamaterial is measured at a distance of 1.5 m from the sound source. The SPL gain (*G*) is obtained from *P*_m_ − *P*_o_ (or *P*_r_ − *P*_o,_ for the case with no metamaterial), where *P*_o_ is the SPL for free space (i.e., no structures are present) measured at the same distance from the source. As expected, the sound field distribution is symmetric with respect to the *y*-axis. Further, the SPL gain measurements along the axis of origin shown in Fig. 3(b) indicate that a distinct focal line forms at *f* ≈ (0, 30) for *ω* = 1490 Hz, where gain ~9 dBi is detected. Figure 3(c,d) show the calculated and measured SPL gain at position (0, 30) with respect to the normally incident *ω* between 1000 and 2000 Hz. Although the acoustic meta-surface converts a propagating wave (PW) into a surface wave (SW) via a dramatic change in d*Φ*(*x*)/d*x* > 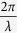 around the 1200 Hz frequency range^37^, an efficient sound collimation is obtained at the *ω* ≈ 1400–2000 Hz region, which is due to the artificially designed phase gradient.

We also measure the polar sound intensity of the omnidirectional microphone at positions (0, 30) and (0, 100), where the maximum and minimum SPL are measured, respectively; the results are shown in Fig. 3(e,f). The measured sound intensity at the focal line (0, 30) indicates a highly directive beam with a 3-dB beam-width, *B* ≈ 55°. On the contrary, if the microphone is located at an off-focal line, e.g., (0, 100), the acoustic receiver yields a small SPL gain, as illustrated in Fig. 3f; (experimental results). Thus, this gradient impeding meta-surface, which is approximately 1.38 times *λ*, provides efficient radiation patterning compared to the dipole-radiation patterning formed by a periodic array of identical Helmholtz resonators (see Fig. S4).

### Gain and 3-dB Beam-width for Designed Acoustic Meta-surface

**Figure 4:**
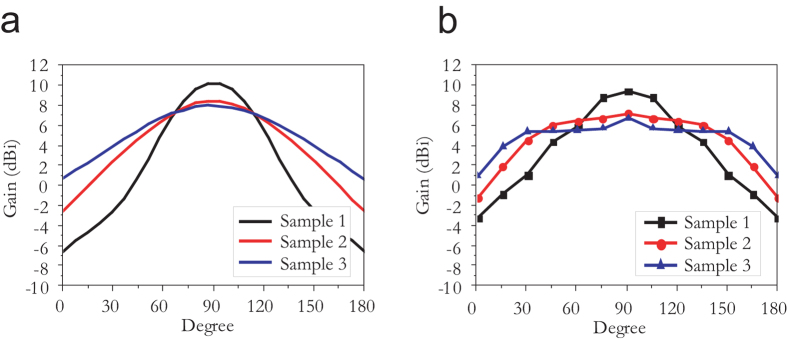
Received beamforming achieved by manipulating the number of acoustic resonators on each side of the acoustic meta-surface, *N*. (**a**) Calculated and (**b**) measured polar sound pressure level (SPL) gain for Samples 1–3, having *N* = 8 (Sample 1), 6 (Sample 2) and 4 (Sample 3), with 1490 Hz incident sound frequency.

We now examine the performance of the sound antenna in the receiving mode with respect to the number of resonators. For instance, the gain and the 3-dB beam-width (*B*) of a parabolic cylinder reflector (such as that shown in Fig. 1(b)) depend on the geometrical parameters and *λ*^25^, such that gain ≈ 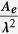 and 3 dB beam-width ≈ 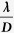, where *A*_e_ is the effective aperture or capture area of the antenna. Similarly, it is also possible to control the gain and directivity by manipulating the total *D* of the acoustic meta-surface. For example, for Samples 2 (*N* = 6 on each side, *D* = 237.5 mm) and 3 (*N* = 4 on each side, *D* = 157.5 mm) at normal incidence (see Fig. S1), the focal lines of the two samples are identically located at (0, 30), similar to that of Sample 1, because of their identical d*Φ*(*x*)/d*x* profiles. However, as illustrated in Fig. 4, Samples 2 and 3 exhibit lower gain (*G*) and directivity because of their smaller *D*, as predicted. In addition, the antenna performance can be also controlled by manipulating *d* (the spacing between the resonators). For instance, if *d* is increased to 30 mm for Sample 1, the d*Φ*(*x*)/d*x* along the meta-surface changes dramatically from that for the original Sample 1 with *d* = 20 mm. This change is due to the smaller *L* resulting from the increased *d* (see Fig. S5). Nevertheless, if we increase *ω* to 1700 Hz, the distinct focal line located at *f* ≈ (0, 120) again yields a highly directive sound antenna with beam-width (*B)* ≈ 30°, because of the small *λ* and large *D*, as illustrated in Figs S5 and S6.

### Sound-Beam Intensity Measurement for Off-axis Receiver

**Figure 5:**
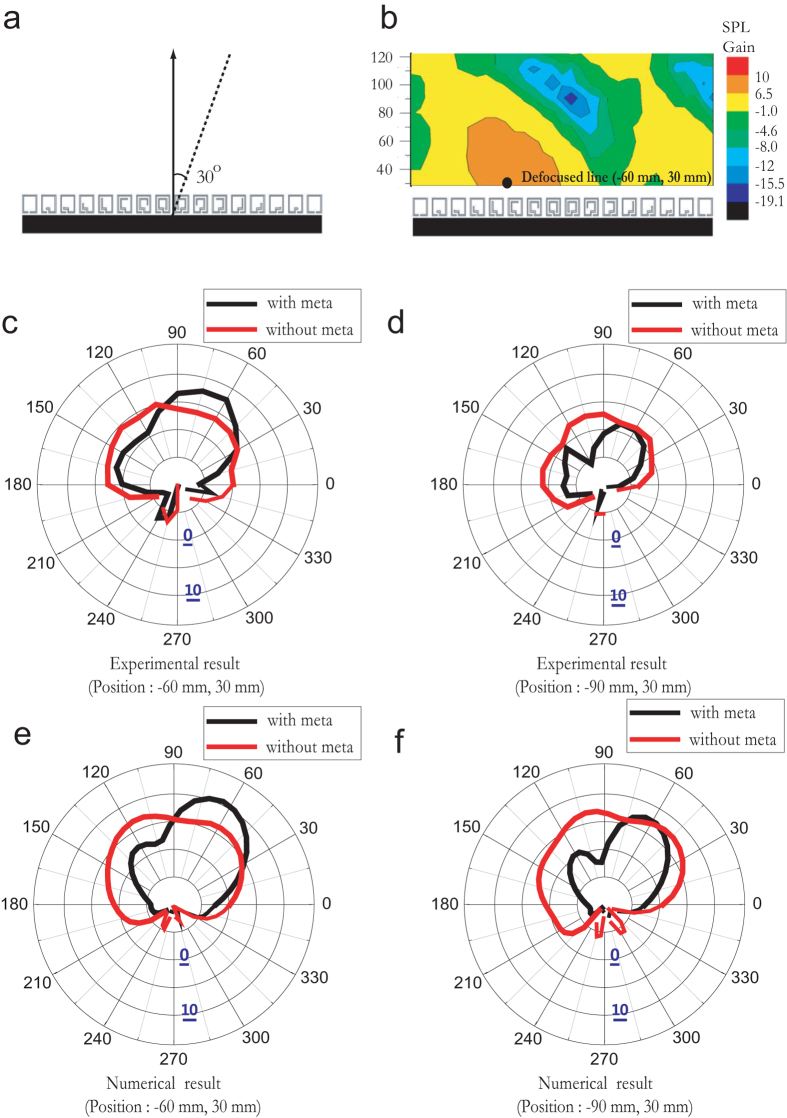
Received beamforming measured by microphone located at off-axis and displaced from focal-line. (**a**) Schematic of experimental setup used to measure sound pressure level (SPL) gain for 30° obliquely incident sound wave at 1490 Hz. (**b**) SPL gain contour for 30° obliquely incident sound wave. Measured polar SPL gain for setup shown in (**a**) at (**c**) (−60, 30) and (**d**) (−90, 30). Calculated polar SPL gain for setup shown in (**a**) at positions (**e**) (−60, 30) and (**f**) (−90, 30). (All positions are in millimetres).

Next, we demonstrate that the location of the acoustic meta-surface defocused line can be manipulated as a function of *θ*. Unlike normally incident sound waves, the obliquely incident plane wave (*θ* = 30°) shown in Fig. 5(a), for example, causes a change in the defocused line location to approximately (−60, 30), as shown in the measured SPL gain map in Fig. 5(b). The experimental polar SPL gain (*G*) of the acoustic signal with and without the meta-surface is also shown for *ω* = 1490 Hz in Fig. 5(c,d) for locations (−60, 30) and (−90, 30), respectively. As expected, if we place the microphone at (−60, 30), the maximum sound intensity at the microphone is measured for an oblique incidence of *θ* = 30° from the normal direction. However, the SPL gain at (−60, 30) is less than that at (0, 30) for the normally incident case, because of phase distortions. This is common for phase array systems in which the SPL gain of the steered beam exhibits cos (θ) dependence. In addition, if the microphone is located at (−90, 30), which is further away from the focal axis, no SPL gain is detected. These experimental results are in close agreement with the numerical results shown in Fig. 5(e,f). The results above show that the textured surface can steer the reflected beam at deviations of up to ±30°, depending on the location of the transmitting or receiving transducers. In addition, this steering angle can be further increased to 45° using an increased number of resonators, with *D* = 517.5 mm (Sample 4, *N* = 13), as shown in Figs S7 and S9.

### Directive Sound Emitters based on Acoustic Meta-surface

**Figure 6:**
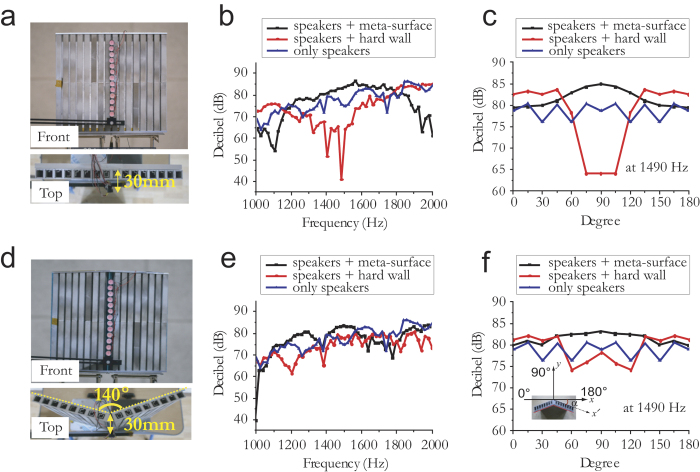
Directive sound antenna in transmitting mode. (**a**) Prototype of flat reflective meta-surface (Sample 1) with phase array feeds consisting of 13 point-like sources. (**b**) Measured microphone sound pressure levels (SPLs) at the normal direction of a plane, which are located 1 m from speakers with a flat reflective surface, a hard-wall reflective surface, and no structure. (**c**) Measured polar SPL for flat surfaces with 1490 Hz speakers. (**d**) Prototype of convex reflective meta-surface Sample 1 with 140° exterior angle and phase array feeds consisting of 13 point-like sources. (**e**) Measured microphone SPLs at the normal direction of a plane, which are located 1 m from sources with a convex reflective meta-surface, convex hard-wall reflective surface, and no structure. (**f**) Measured polar SPL for convex surfaces with 1490 Hz speakers. Inset: The *x*′ axis indicates the direction with tilt angle *α* relative to the *x*-axis.

Finally, we consider a case where the source and receiver are interchanged. In that case, the omnidirectional sound signal originating from a small source located at the focal line bounces from the acoustic meta-surface and is transformed into a highly directional signal via acoustic reciprocity. Figure 6(a) shows that the flat acoustic meta-surface, together with an array feed consisting of 13 periodically aligned point-like speakers with 15-mm spacing, yields a directional sound speaker in the azimuthal direction. Here, each speaker located at (0, 30) generates an almost identical amplitude and phase, thus creating a near-line sound source. In Fig. 6(b), we show the SPL of a microphone located at the normal direction relative to the flat surface. Similar to the flat receiver, a significant SPL gain is observed for the sound speaker for *ω* between 1200 and 1800 Hz. Note that directional beamforming in a transmitting mode is confirmed by the results of the polar SPL measurement at 1490 Hz, which are shown in Fig. 6(c). In addition, sound beamforming can also be obtained using a variety of complex surfaces, such as convex, flexible, or conformal surfaces, with the aid of reshaped phase reflection. In Fig. 6(d), we demonstrate a directive sound speaker with a convex meta-surface corresponding to Sample 1 with an exterior angle of 140°. If we consider the convex surface with tilted angle α, the redesigned reflection phase 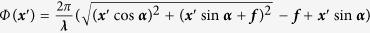 due to the α at the *x*-axis yields a highly directional sound antenna, where the *x*′ axis indicates the direction with tilted angle α relative to the *x*-axis (see Supplementary Fig. S10). Because of the small effective area and the phase change of the tilted angle α, the SPL gain of the convex meta-surface is smaller than that of the flat meta-surface. However, this surface also provides directive beamforming with an SPL gain of more than 7 dBi at 1490 Hz, as shown in Fig. 6(e,f).

## Discussion

According to the Huygens-Fresnel Principle, the directivity is fundamentally limited by the physical size of the source relative to the *λ*. More directional sound beamforming can be achieved using a large speaker or an array of small speakers. Here, the inherent physical limitation of a small source (or receiver) can be overcome by utilizing a planar acoustic meta-surface. Our experimental and theoretical results essentially confirm that the use of a gradient-impeding meta-surface generates new boundary conditions that allow the reflection phase to be manipulated at will. In contrast to the mechanisms employed in conventional directional antennas such as horns, the acoustic meta-surface controls the directional radiation patterning reflected from a complex surface, such as a convex, flexible, or conformal surface. To further enhance the directivity (or gain), various forms of sub-wavelength Helmholtz resonator arrays, with different sizes, dimensions, and configurations, can be used to tune the reflection phases along the acoustic meta-surface. In our experiment, we utilized eight different Helmholtz resonators with different coiling paths, each operating as a different phase shifter and reflector. If an increased number of resonator elements are employed, pencil-like beamforming can be obtained via a large effective aperture with optimally designed coiling paths. Similar to electromagnetic parabolic antenna, beam steering with high gain can be achieved by repositioning the acoustic transducers off axis, i.e., displaced from the focal line. Furthermore, dynamic beam steering is also possible with an active phase array, where each phase element has a different phase. Thus, these beamforming techniques will lead to promising applications in fields such as acoustic imaging, sonic weaponry, and underwater communication.

## Method

### Acoustic meta-surface preparation

The Helmholtz resonators were manufactured via computer numerical control (CNC) precision machining. In order to create the parabolic reflection phase along the flat surface, each Helmholtz resonator (with a unique coiling path) was inserted within a prefabricated aluminium jig precision machining, so as to yield a desired *g* = 2 mm between the hard walls and acoustic meta-surface.

### Numerical simulation of gradient-impeding acoustic meta-surfaces

The finite element method (FEM) based on COMSOL multi-physics software was employed for the scattered acoustic field simulation in Fig. 2(f–h). Perfectly matched layers (PMLs) were imposed on the outside of the boundary in the simulation domain in order to eliminate the reflection.

### Reflection phase calculation

In order to calculate the *Φ*(*x*) difference depending on the resonators (which had varying coiling path lengths), we compared the reflection coefficients of the rigid walls with and without metamaterials utilizing COMSOL multi-physics software. The reflection coefficients were calculated using normally incident sound waves. Here, we analysed 2D single unit cells with periodic boundary conditions and employed perfectly matched layer (PML) absorbing boundary conditions.

### SPL measurement of directive sound receivers

A plane-like sound wave was generated by a BP 012 omnidirectional loudspeaker connected to an Agilent 33522A function generator. The SPL was measured using a G.R.A.S 46BE 1/4-inch microphone located at the focal position above the gradient-impeding surface. To measure the polar SPL *G* depending on the *θ* of the incident sound wave, the directional meta-surface was progressively rotated in 15° increments.

### SPL measurement of directive sound emitters

A point-like sound wave was generated by a phase array feed consisting of three CM-23299-00 8.38 × 16.54 MM speakers connected by an Agilent 33522A function generator. The SPL was measured by a G.R.A.S 46BE 1/4-inch microphone positioned 1 m from the acoustic meta-surface. To obtain the polar SPL, the SPL was measured by progressively rotating the microphone in 15° increments.

## Additional Information

**How to cite this article**: Song, K. *et al*. Directional Reflective Surface Formed via Gradient-Impeding Acoustic Meta-Surfaces. *Sci. Rep.* **6**, 32300; doi: 10.1038/srep32300 (2016).

## Supplementary Material

Supplementary Information
