# Supplementary material for: Directional Reflective Surface Formed via Gradient-Impeding Acoustic Meta-Surfaces
Source: Sci Rep. 2016 Aug 26;6:32300. doi: 10.1038/srep32300 (PMC4999803; doi:10.1038/srep32300)
Supplement: Supplementary Information [file srep32300-s1.pdf]

Supplementary Information for Directional Reflective Surface Formed via Gradient-Impeding Acoustic Meta-surfaces

Kyungjun Song[1*], Jedo Kim[2], Hur Shin[1], Jun-Hyuk Kwak[1], Seong-Hyun Lee[3],Taesung Kim[4]

[1]Department of Nanoconvergence Systems, Korea Institute of Machinery and Materials, 156 Gajeongbuk-Ro, Daejeon, 305-343, Republic of Korea

[2]Department of Mechanical System Design Engineering, Hongik Univ. 94 Wausan-ro, Mapo-gu, Seoul 121-791, Seoul, Republic of Korea

[3]Department of System Dynamics, Korea Institute of Machinery and Materials, 156 Gajeongbuk-Ro, Daejoen, 305-343, Republic of Korea

[4]Department of Mechanical Engineering, Ulsan National Institute of Science and Technology (UNIST), 50 UNIST-gil, Ulsan 44919, Republic of Korea.

Correspondence : Kyungjun Song (email: songk@kimm.re.kr)

Supplementary Information

Supplementary Note

Note 1: Radiation patterning of artificially textured surface with periodic array of identical Helmholtz resonators backed by rigid hard wall

Let us consider sound beamforming obtained using acoustic metamaterials consisting of a linear array of identical Helmholtz resonators with p = 36 mm placed at distance d = 20 mm above the rigid walls, as illustrated in Fig. S4(a). The sound pressure P is simply obtained using the method of images illustrated in Fig. S4(b). If the point source is located at a distance h from the rigid wall with acoustic meta-materials, the pressure field of the point source can be described by

$$P_i = A\left(\frac{1}{r_-}e^{-jk\,r_-}\right) \tag{S1}$$

where $r\_$ is the distance from the point (0, h). The reflected sound pressure $\boldsymbol{P}_{\mathrm{r}}$ has the same magnitude and reflection phase Φ, because the Helmholtz resonator yields the Φ induced by the impedance of a series LC resonator. Based on the image method, a second source of the same strength and with a different reflection phase Φ is placed at (0, -h), thus yielding the reflected sound pressure

$$\boldsymbol{P_r = A\left(\frac{1}{r_+}e^{-jk\,r_+ -j\Phi}\right)} \tag{S2}$$

where r_+ is the distance from the point (0, -h). Thus, the p in the region z > 0 is given by the sum of the incident pressure p_i and p_{r}

$$\boldsymbol{P = P_i + P_r = A\left(\frac{1}{r_-}e^{-jk\,r_-} + \frac{1}{r_+}e^{-jk\,r_+ -j\Phi}\right)} \tag{S3}$$

where Φ is the reflectance phase of the rigid walls with the acoustic meta-surfaces. In the approximation r >> h, Eq. (S3) becomes

$$\boldsymbol{P = \frac{A}{r}e^{-jkr}\left(\frac{e^{jk\,\Delta r}}{1-\frac{\Delta r}{r}} + \frac{e^{-jk\,\Delta r - j\Phi}}{1+\frac{\Delta r}{r}}\right)} \tag{S4}$$

where $\Delta r \sim h cos\theta$. Because $\frac{\Delta r}{r} \ll \mathbf{1}$, Eq. (S4) becomes

$$\boldsymbol{P} = \frac{\boldsymbol{A}}{\boldsymbol{r}} \boldsymbol{e}^{-jkr} \left(\boldsymbol{e}^{jkh\cos\theta} + \boldsymbol{e}^{-jkh\cos\theta - j\Phi} \right) \tag{S5}$$

For example, the rigid boundary at $\Phi = 0$ generates a reflected wave with the same magnitude and phase as the incident field. On the other hand, the pressure release boundary condition of $\Phi = \pi$ yields a reflected wave with the same magnitude but the opposite phase to the incident field. Fig. S4(c) illustrates the reflection phase of a single unit cell with $p = 36$ mm, calculated using the finite element method (FEM). The artificial textured surface at 1200 Hz provides the pressure release boundary condition at $\Phi = \pi$. The numerical and experimental results for the radiation patterning obtained for a microphone located at $h = 70$ mm are illustrated in Fig. S4(d) and (e), respectively. Thus, the sound intensity at 1200 Hz provides dipole radiation resulting from the textured meta-surfaces.

Note 2 : **Radiation bandwidth for array of identical Helmholtz resonators backed by rigid hard wall**

Let us attempt to calculate the radiation bandwidth (BW) for an array of identical Helmholtz resonators backed by a rigid hard wall. In the case of the array of Helmoholtz resonators, the electrical analogue can be described by series impedance Z = $\eta + j\omega L + 1/j\omega C$ where the sound pressure is regarded as a voltage source. The amount power dissipation in the radiation is measure of the radiation efficiency. Thus, the radiation efficiency is at a maximum at $\Phi = \pi$ (at resonance frequency), which means that the textured surface acts as a low-impedance boundary. The radiation efficiency of the textured surface changes according to the variation of the surface impedance Z_m. Here, the radiation bandwidth (BW) indicates the distance between the two frequencies when the radiation falls to half its maximum value. These conditions occur when the magnitude of the textured surface Z_m is equal to the characteristic impedance of air η

$$|Z_m| = \eta. \tag{S6}$$

Substituting $Z_m = j\omega L + 1/j\omega C$ into Eq. (S6), we obtain

$$|j\omega L + 1/j\omega C| = \eta. \tag{S7}$$

Through numerical derivations, a detailed expression for ω can be obtained, where

$$\omega^2 = \frac{1}{LC} + \frac{\eta^2}{2L^2} \pm \frac{\eta}{L}\sqrt{\frac{1}{LC} + \frac{\eta^2}{4L}}. \tag{S8}$$

Discarding the $\left(\frac{\eta}{L}\right)^2$ term in Eq. (S8), because $\frac{1}{LC}$ >> $\left(\frac{\eta}{L}\right)^2$, we find

$$\omega \sim \omega_o \sqrt{1 + \eta\sqrt{\frac{C}{L}}} \sim \omega_o \left[1 \pm \frac{\eta}{2}\sqrt{\frac{C}{L}}\right], \tag{S9}$$

where ω_0 is the resonant frequency. Thus, the radiation bandwidth (BW) of the array of identical Helmholtz resonators, which consists of a series of LC resonators, can be expressed as

$$BW \sim \frac{\Delta\omega}{\omega_0} \sim \eta\sqrt{\frac{C}{L}} \ . \tag{S10}$$

From Eq. (S10), the radiation bandwidth is controlled by the geometry of the Helmholtz resonators and the environmental materials, such as air or water.

Supplementary Figures

Figure S1

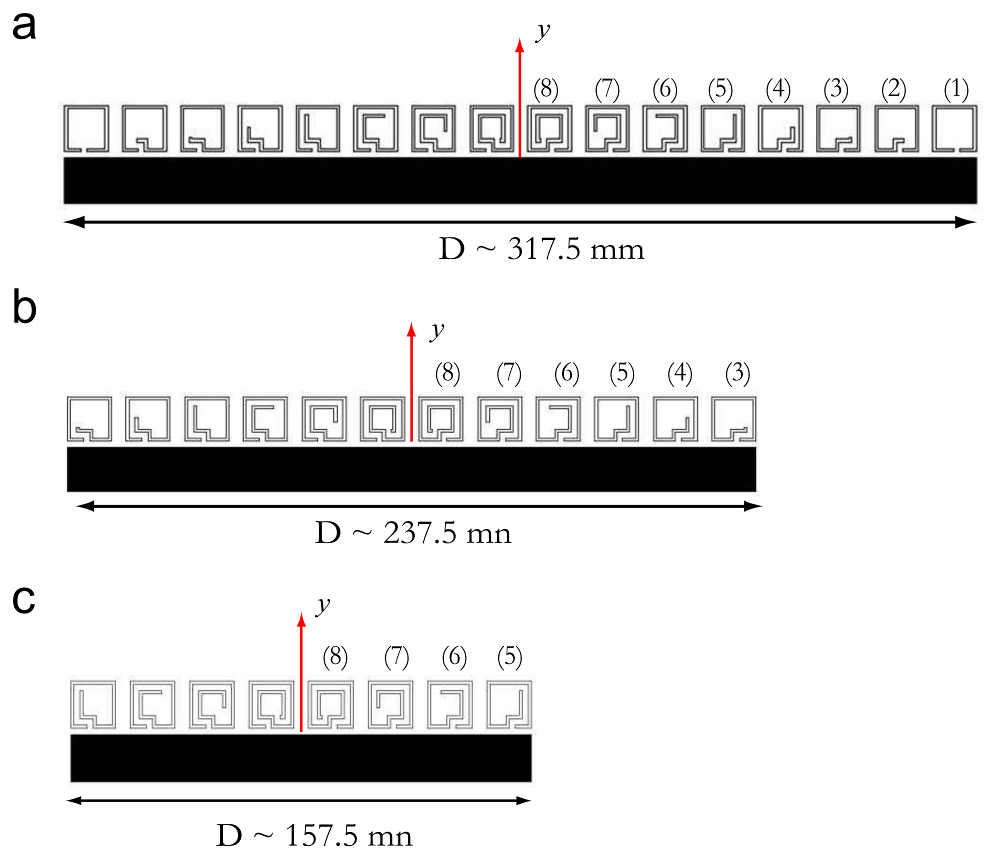

Supplementary Fig. S1 | Basic schematics of Samples 1, 2 and 3. Basic schematic of meta-surfaces consisting of a periodic array of Helmholtz resonators with different coiling paths: (a) Sample 1, the number of resonator on each side, N, is 8, (b) Sample 2, N=6, and (c) Sample 3, $N = 4$.

Figure S2

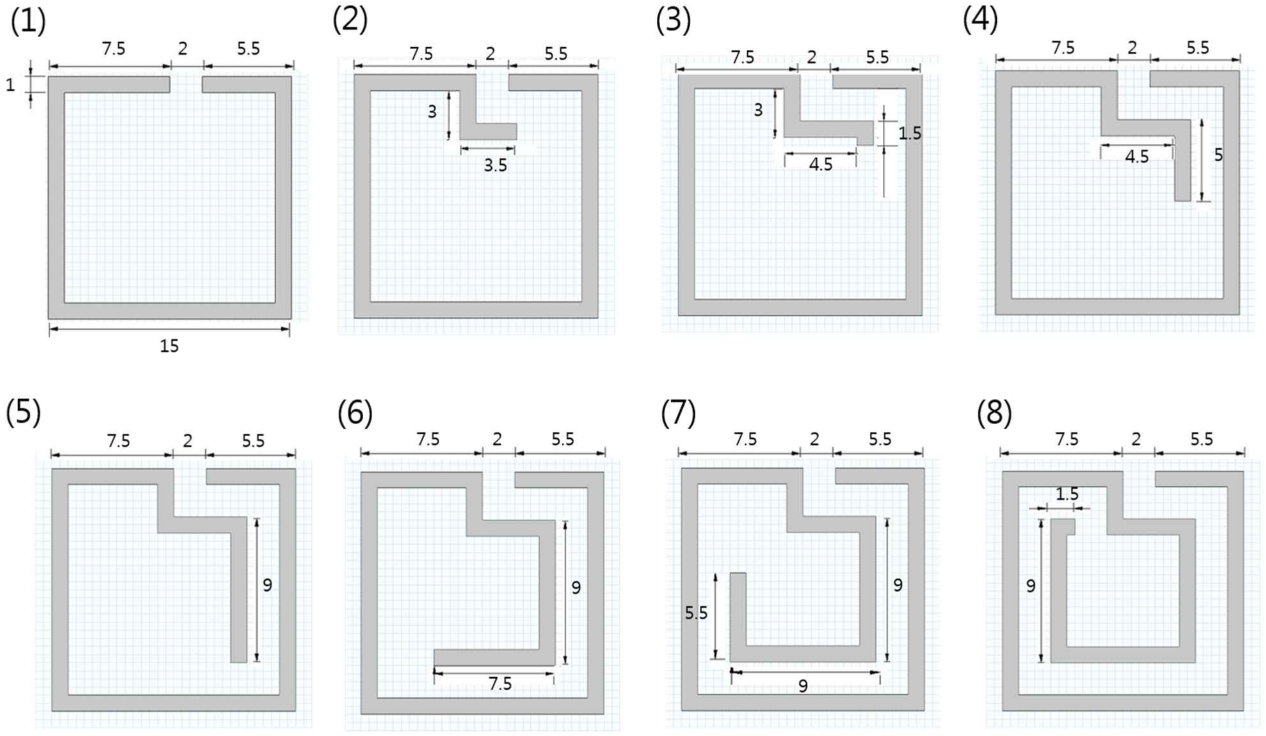

Supplementary Fig. S2 | Basic geometries of 8 different Helmholtz resonator elements.

Figure S3

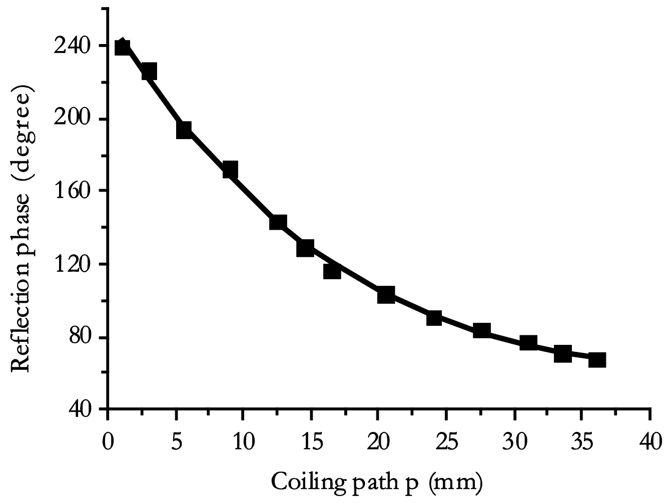

Supplementary Fig. S3 | Plot of reflection phase *Φ* versus coiling path at 1490Hz. Points show the result of FEM calculations, and line is given by Eqn.(3).

Figure S4

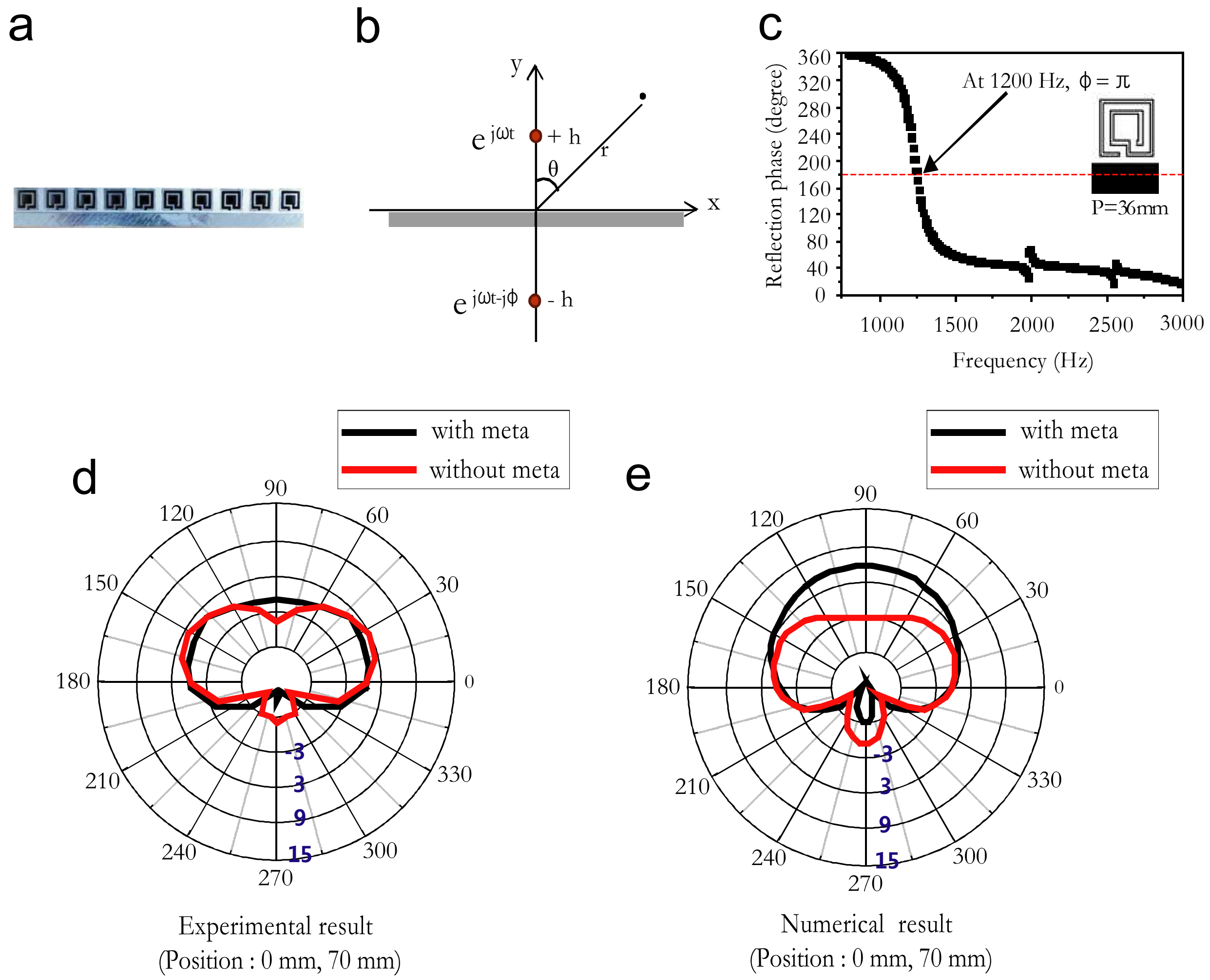

Supplementary Fig. S4 | Radiation patterning for periodic array of identical Helmholtz resonator arrays backed by rigid surface. (a) The sample has periodic distance d = 20 mm and path length p = 36 mm. The total number of Helmholtz resonators is 10. (b) Image method for calculating the acoustic field of an acoustic source near an artificial textured surface above a rigid wall. The source and source image are located at (0, h) and (0, -h), respectively. The field point is located at ($r sin\theta$, $r cos\theta$). (c) Reflection phase of periodic array of identical Helmholtz resonators calculated via finite element method (FEM). (d) Experimental results for polar sound intensity at (0, 70) for 1200Hz frequency. (e) Numerical results for polar sound intensity at (0, 70), for 1200Hz frequency.

Figure S5

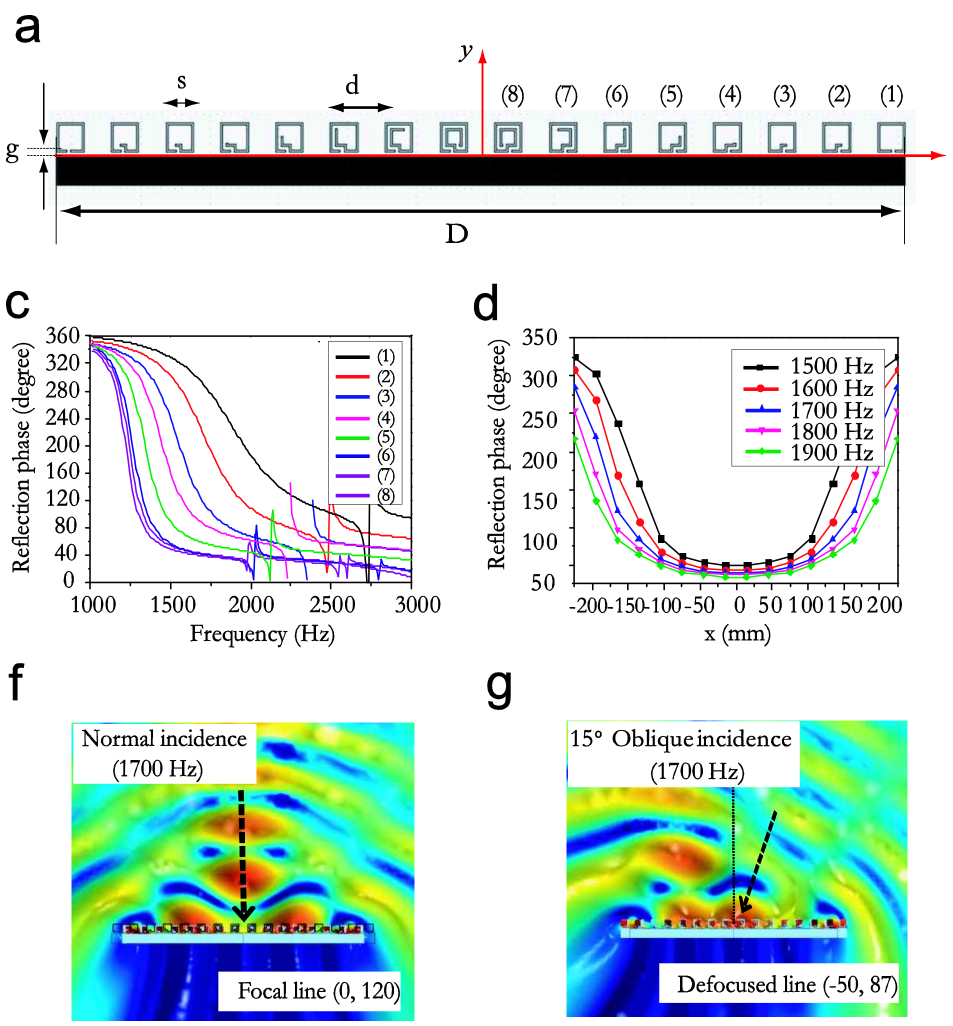

Supplementary Fig. S5 | (a) Schematic of acoustic meta-surface consisting of array of Helmholtz resonators with varying coiled path lengths, p (Sample 1, number of resonators on each side, $N = 8$). The subwavelength width, s, subwavelength separation, d, gap, g, and total length, D, are fixed to 15, 30, 2, and 300 mm, respectively, while p is adjusted to 1, 5.5, 9, 12.5, 16.5, 24, 31, and 36 mm. (b) Calculated reflection phase $\Phi(x)$ for resonators having varying p, which comprise the meta-surface. (c) Calculated $\Phi(x)$ for meta-surface at various incident sound frequencies. Sound pressure level (SPL) profile for (f) normally incident plane wave and (g) 15° obliquely incident plane wave at 1700 Hz resulting in focal points at approximately (0, 120) and approximately (-50, 87), respectively. (All positions are in millimetres.)

Figure S6

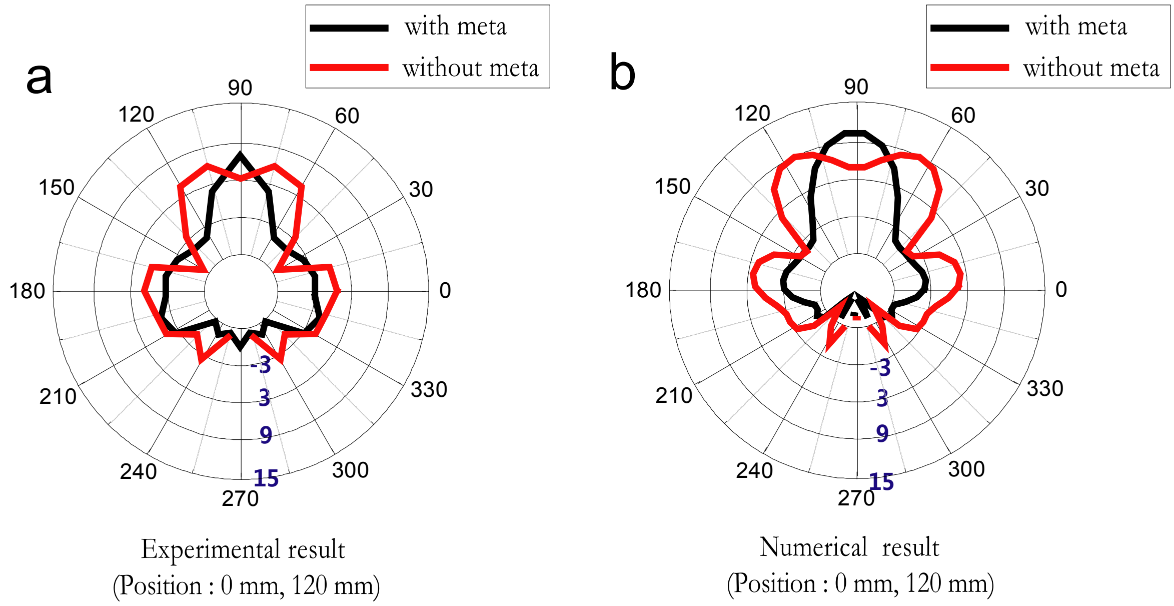

Supplementary Fig. S6 | Sample 1 prototype with subwavelength separation *d* = 30 mm. At 1700 Hz, experimental (a) and numerical result (b) for polar sound pressure level (SPL) gain for sound wave at (0, 120). (All positions are in millimetres.)

Figure S7

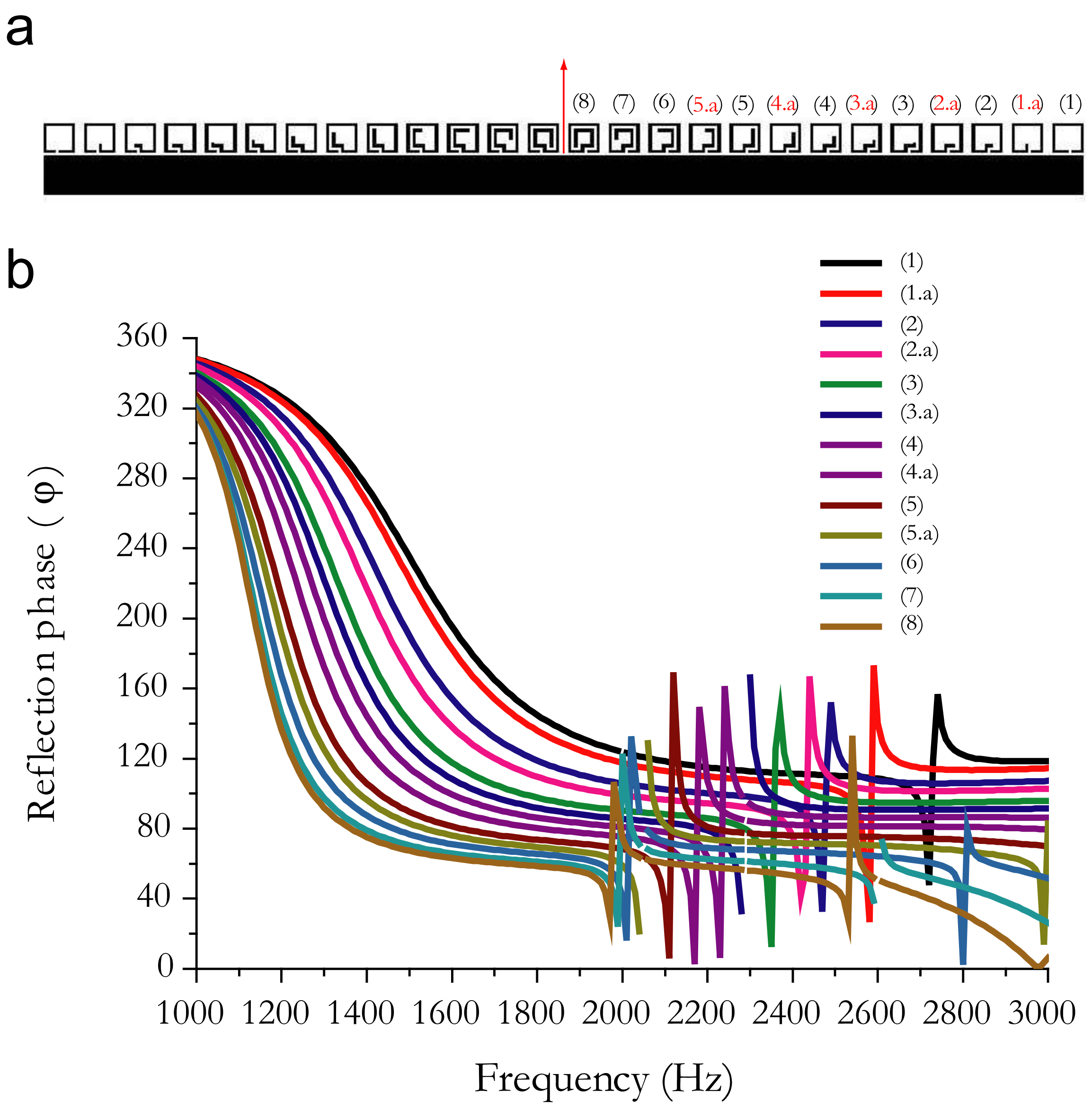

Supplementary Fig. S7 | (a) Schematic of acoustic meta-surface consisting of array of Helmholtz resonators with varying coiled path lengths, *p* (Sample 4, number of resonators on each side, $N = 13$). The subwavelength width, *s*, subwavelength separation, *d*, gap, *g*, and total length, *D*, are fixed to 15, 20, 2, and 517.5 mm, respectively, while *p* is adjusted to 1, 3, 5.5, 9, 12.5, 14.5, 16.5, 20.5, 24, 27.5, 31, 33.5 and 36 mm. (b) Calculated reflection phase $\Phi(x)$ for resonators having varying *p*, which comprise the meta-surface.

Figure S8

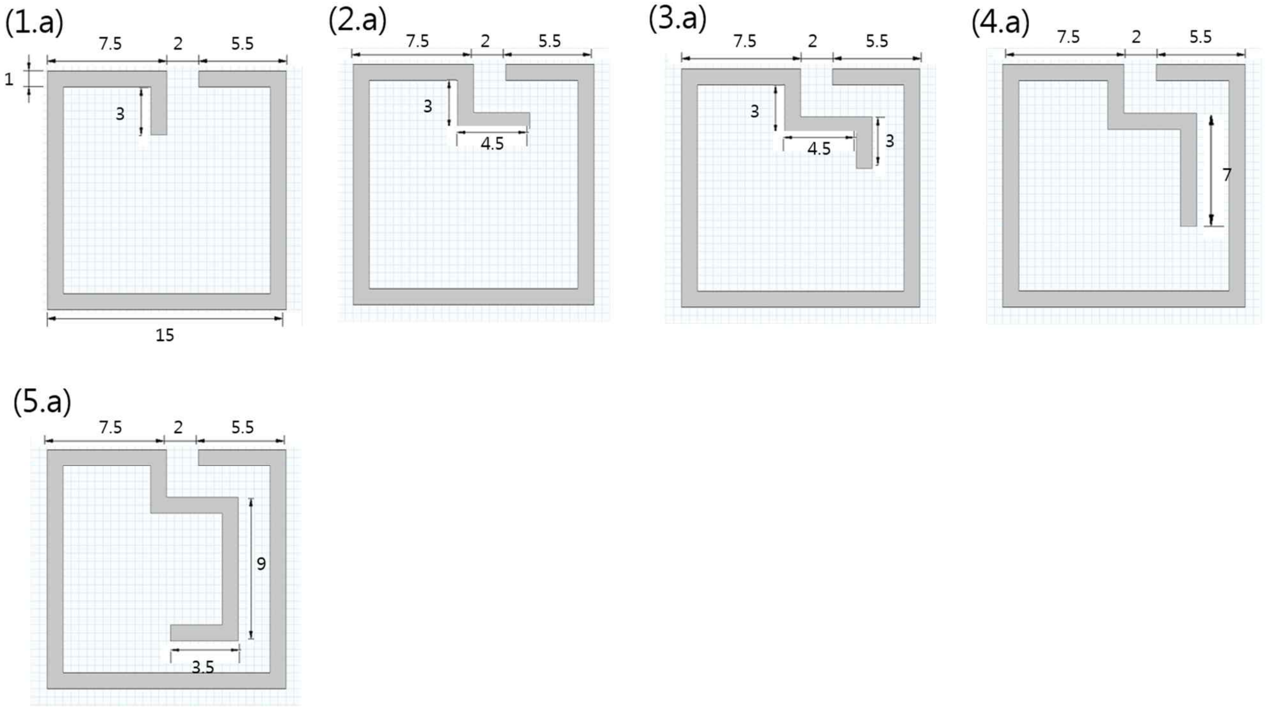

Supplementary Fig. S8 | Basic geometries of 5 additional Helmholtz resonators

Figure S9

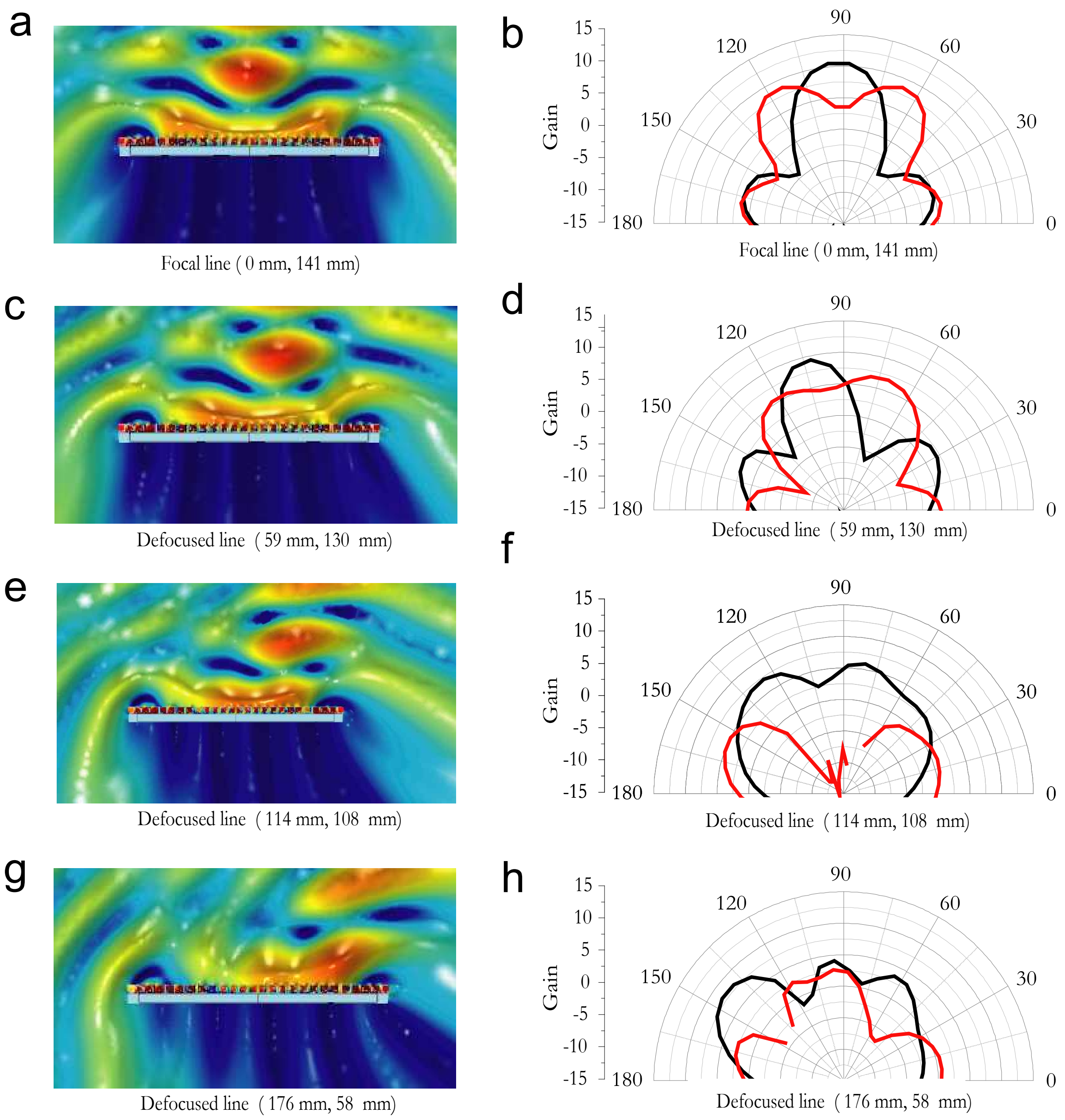

Supplementary Fig. S9 | Numerical Simulations. (a, c, e, g) At 1490 Hz, sound pressure level results for Sample 4 for normally, 15° obliquely, 30° obliquely, and 45° obliquely incident plane waves, respectively. (b, d, f, h) At 1490 Hz, calculated polar sound pressure level (SPL) gain for Sample 4 depending on detector positions: (0, 141), (59, 130), (114, 108), and (176, 58), respectively. (All positions are in millimetres.)

Figure S10

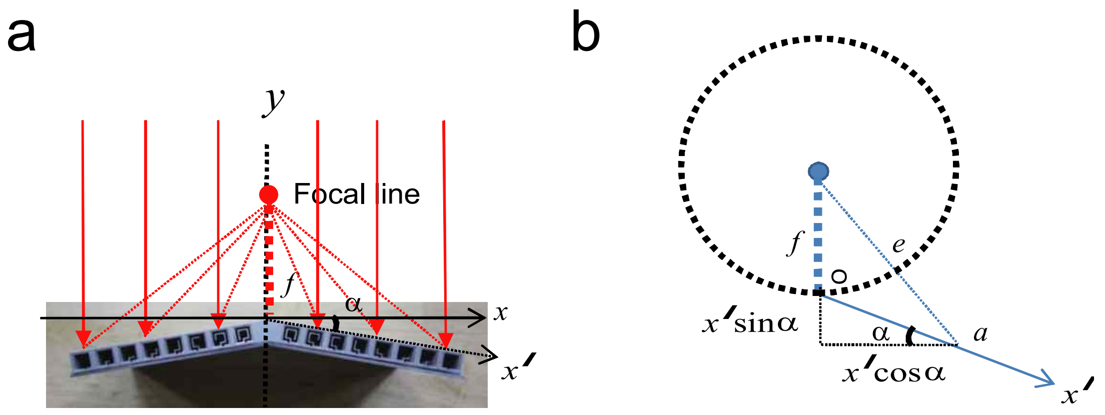

Supplementary Fig. S10 | Convex acoustic metamaterial antenna design. (a) Conceptual figures of convex directional surface with symmetric phase gradient $d\Phi(x')/dx'$ with respect to the origin. (b) The phase shift of the x' direction with tilt angle α relative to the x-axis can be obtained by multiplying the distance $\overline{ae}$ by $2\pi/\lambda$.

Figure S11

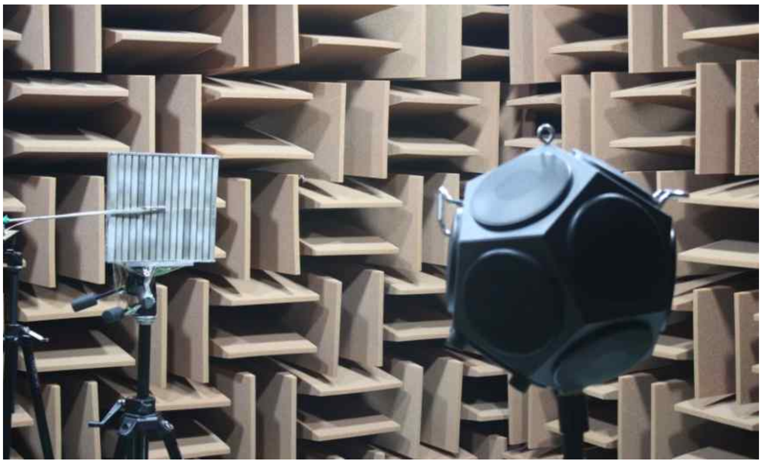

Supplementary Fig. S11 | Photograph of experimental setup of sound antenna in a receiving mode for measuring microphone sound pressure level above the meta-surface 1.5 m away from sound speaker.

Figure S12

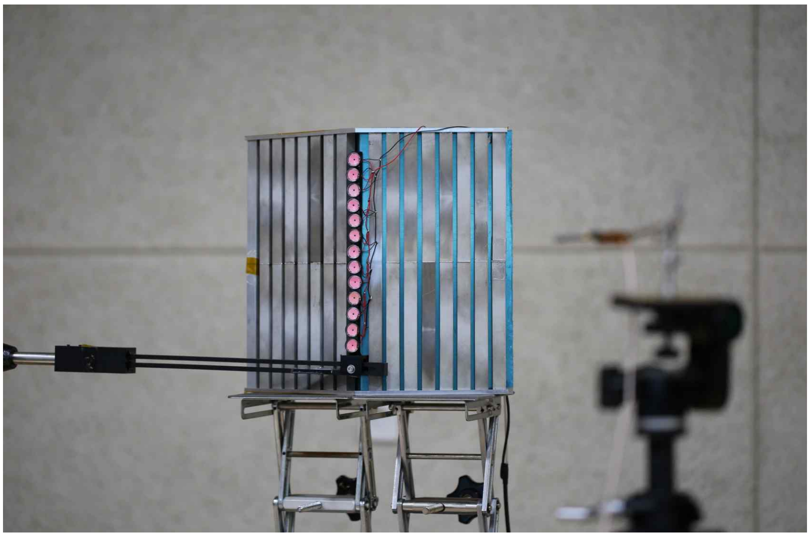

Supplementary Fig. S12 | Photograph of experimental setup of sound antenna in a transmitting mode for measuring microphone sound pressure level above the meta-surface 1 m away from sound speaker.
